# Secretogranin II; a Protein Increased in the Myocardium and Circulation in Heart Failure with Cardioprotective Properties

**DOI:** 10.1371/journal.pone.0037401

**Published:** 2012-05-24

**Authors:** Helge Røsjø, Mats Stridsberg, Geir Florholmen, Kåre-Olav Stensløkken, Anett Hellebø Ottesen, Ivar Sjaastad, Cathrine Husberg, Mai Britt Dahl, Erik Øie, William E. Louch, Torbjørn Omland, Geir Christensen

**Affiliations:** 1 Division of Medicine, Akershus University Hospital, Lørenskog, Norway; 2 Center for Heart Failure Research and K.G. Jebsen Cardiac Research Centre, Institute of Clinical Medicine, University of Oslo, Oslo, Norway; 3 Department of Medical Sciences, Uppsala University, Uppsala, Sweden; 4 Institute for Experimental Medical Research, Oslo University Hospital, Ullevål, Oslo, Norway; 5 Department of Molecular Biosciences, University of Oslo, Oslo, Norway; 6 Department of Clinical Molecular Biology, Akershus University Hospital, Lørenskog, Norway; 7 Research Institute for Internal Medicine, Oslo University Hospital, Rikshospitalet, Oslo, Norway; Virginia Commonwealth University Medical Center, United States of America

## Abstract

**Background:**

Several beneficial effects have been demonstrated for secretogranin II (SgII) in non-cardiac tissue. As cardiac production of chromogranin A and B, two related proteins, is increased in heart failure (HF), we hypothesized that SgII could play a role in cardiovascular pathophysiology.

**Methodology/Principal Findings:**

SgII production was characterized in a post-myocardial infarction heart failure (HF) mouse model, functional properties explored in experimental models, and circulating levels measured in mice and patients with stable HF of moderate severity. SgII mRNA levels were 10.5 fold upregulated in the left ventricle (LV) of animals with myocardial infarction and HF (p<0.001 vs. sham-operated animals). SgII protein levels were also increased in the LV, but not in other organs investigated. SgII was produced in several cell types in the myocardium and cardiomyocyte synthesis of SgII was potently induced by transforming growth factor-β and norepinephrine stimulation *in vitro*. Processing of SgII to shorter peptides was enhanced in the failing myocardium due to increased levels of the proteases PC1/3 and PC2 and circulating SgII levels were increased in mice with HF. Examining a pathophysiological role of SgII in the initial phase of post-infarction HF, the SgII fragment secretoneurin reduced myocardial ischemia-reperfusion injury and cardiomyocyte apoptosis by 30% and rapidly increased cardiomyocyte Erk1/2 and Stat3 phosphorylation. SgII levels were also higher in patients with stable, chronic HF compared to age- and gender-matched control subjects: median 0.16 (Q1–3 0.14–0.18) vs. 0.12 (0.10–0.14) nmol/L, p<0.001.

**Conclusions:**

We demonstrate increased myocardial SgII production and processing in the LV in animals with myocardial infarction and HF, which could be beneficial as the SgII fragment secretoneurin protects from ischemia-reperfusion injury and cardiomyocyte apoptosis. Circulating SgII levels are also increased in patients with chronic, stable HF and may represent a new cardiac biomarker.

## Introduction

Heart failure (HF) is associated with changes in systemic and pulmonary hemodynamics, complex neurohumoral activation, as well as local molecular alterations in the myocardium [1]. Given that coronary artery disease is a leading cause of HF [1], a better understanding of the pathophysiology of myocardial ischemia and HF development is needed. Regulation of hormonal factors in the failing myocardium can directly affect function and survival of cardiac cells [1], and result in increased circulating levels of proteins secreted from the myocardium [2]. Thus, by identifying proteins that are regulated in the failing myocardium, we may enhance our understanding of the pathophysiology of HF and discover new diagnostic and prognostic HF biomarkers.

The protein secretogranin II (SgII) is a 587 amino acid long protein from the chromogranin-secretogranin (granin) protein family [3]. Two other members of the granin protein family, chromogranin (Cg) A and B have been found increased in HF [4]–[6], and may represent novel cardiac biomarkers [4]–[9]. In addition, chromogranins may affect myocardial function in HF [10], [11]. For SgII, functional aspects have mainly been attributed to the short 33 amino acid peptide secretoneurin (SgII_154–186_) and a number of interesting pathophysiological effects have been reported in other organs than the heart [3]. SgII production is increased by hypoxia in skeletal muscle [12], and secretoneurin protects against apoptosis and ischemic injury in the brain and skeletal muscle [13], [14]. The proteases PC1/3 and PC2 have been identified as the principal proteases for processing of SgII to shorter fragments [15], [16]. As other granin proteins appear to be upregulated during HF development, we hypothesized that SgII production is increased in the myocardium and circulation in HF, and that SgII could play a role in the pathophysiology of HF following myocardial ischemia.

## Results

### Left Ventricular SgII Gene Expression is Upregulated During HF Development

To study SgII production in the left ventricle (LV) in HF, we first compared SgII mRNA levels in non-infarcted LV tissue of HF animals to levels in sham animals. HF animals exhibited increased lung weights and increased LV and right ventricular mass, reflecting pulmonary congestion and compensatory myocardial hypertrophy (Table 1). By echocardiography, the HF animals also had marked LV dilatation and increased left atrial size compared to sham animals (Table 1). Furthermore, mass spectrometry peptide mass fingerprinting of a very strong band in Commassie stained gels from pulmonary tissue of HF mice identified albumin, which is a testament to the pronounced congestion and leakage of proteins into the lungs of our HF mice (Supporting Information S1).

**Table 1:** Descriptive statistics of animals.

	Sham(n = 21)	HF(n = 27)	p
Animal weight, day 0 (g)	24.4±0.4	24.4±0.4	0.95
Lung weight/tibial length (g/mm)	0.079±0.001	0.156±0.007	<0.001
LV mass/tibial length (g/mm)	0.045±0.001	0.058±0.001	<0.001
RV mass/tibial length (g/mm)	0.011±0.001	0.013±0.001	0.01
LV CgA mRNA levels	1.0±0.4	4.8±1.4	0.02
LV CgB mRNA levels	1.0±0.1	5.2±0.7	<0.001
LV BNP mRNA levels	1.0±0.1	5.8±0.7	<0.001
IVSd (mm)	0.8±0.1	0.3±0.0	<0.001
IVSs (mm)	1.2±0.1	0.4±0.0	<0.001
LVDd (mm)	3.7±0.2	6.0±0.1	<0.001
LVDs (mm)	2.4±0.2	5.5±0.1	<0.001
LVFS (%)	18±6	8±1	0.11
PWd (mm)	0.8±0.1	0.7±0.0	0.37
PWs (mm)	1.1±0.1	1.0±0.1	0.32
LAD (mm)	1.6±0.1	2.7±0.1	<0.001

LV indicates left ventricle; RV, right ventricle; CgA, chromogranin A; CgB, chromogranin B; BNP, B-type natriuretic peptide; IVSd, intraventricular septum thickness in diastole; IVSs, intraventricular septum thickness in systole; LVDd, LV diameter in diastole; LVDs, LV diameter in systole; LVFS, LV fractional shortening; PWd, posterior wall thickness in diastole; PWs, posterior wall thickness in systole; and LAD, left atrial diameter.

mRNA levels were investigated in a subset of animals (n = 9 HF, n = 8 sham) and are presented as fold change±SEM. Echocardiographic data are reported as mean±SEM and are obtained from a representative subset of animals (13 HF animals, 6 sham animals).

**Figure 1:**
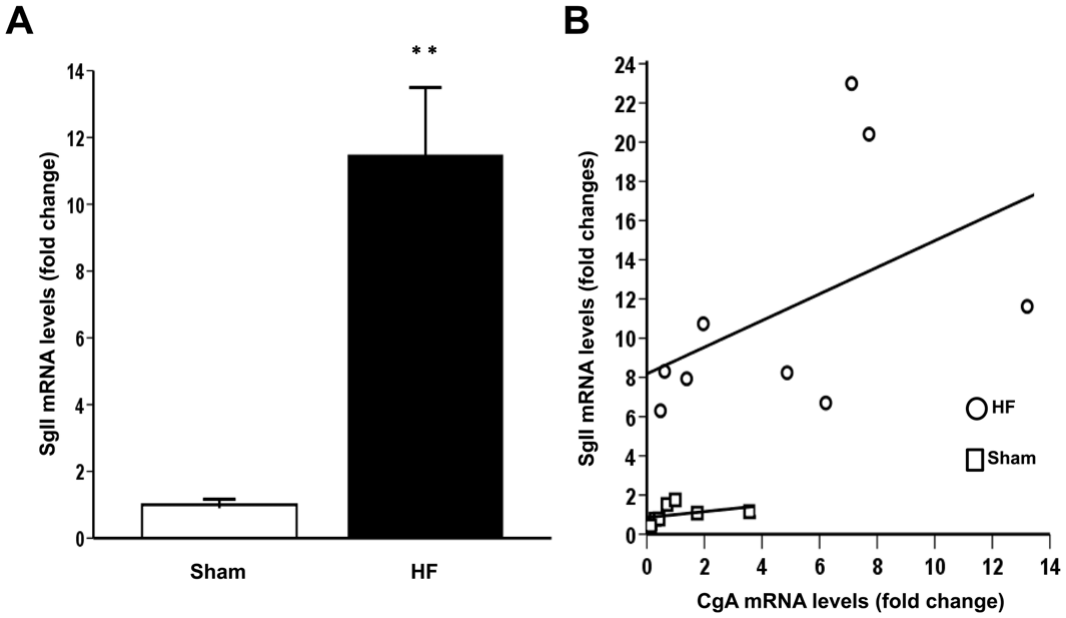
Left ventricular SgII gene expression in heart failure. A, SgII mRNA levels in non-infarcted left ventricular tissue during HF development. SgII mRNA levels were 10.5 fold increased (p<0.001) in non-infarcted LV tissue in HF animals (n = 9) compared to sham-operated animals (n = 8). Gene expression was measured by RT-qPCR and is presented as fold change ± SEM. B, LV SgII mRNA levels were closely correlated with CgA mRNA levels in both HF (r = 0.68, p = 0.04) and sham animals (r = 0.81, p = 0.02). **p<0.001.

LV SgII mRNA levels were markedly upregulated in HF animals compared to sham animals (10.5 fold increase, p<0.001, Figure 1A). This was a greater relative increase than observed for B-type natriuretic peptide (BNP), CgA, or CgB mRNA levels (Table 1). SgII mRNA levels correlated significantly with CgA mRNA levels in HF (r = 0.68, p = 0.04, Figure 1B), but not with CgB or BNP mRNA levels (Supporting Information Table S1).

### SgII Production is Increased in the LV in HF

**Figure 2:**
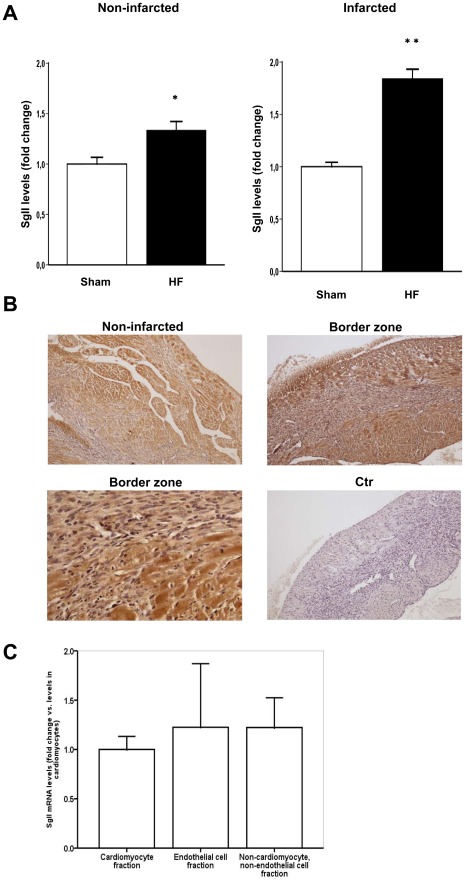
SgII is produced by cardiomyocytes and increased in the left ventricle during HF development. A, SgII protein levels as measured by RIA were increased in both the non-infarcted and infarcted region of the LV in HF animals compared to levels in the myocardium of sham-operated animals (n = 9 for both groups). B, Representative photomicrographs of myocardial tissue sections of a HF mouse demonstrating SgII immunoreactivity (brown staining) in cardiomyocytes of the non-infarcted LV (upper left image). Images of the area bordering the infarcted zone (border zone) are presented in the upper right image (magnification: ×100) and the lower left image (magnification: ×400) and demonstrate SgII immunoreactivity also in non-cardiomyocyte cardiac cells, including fibroblasts. In the upper right image, the infarct area is seen on the left side with granulation tissue in between non-infarcted tissue in the center. Bottom right picture demonstrates very weak staining after use of non-immune rabbit serum as control (ctr). Magnification: ×100 except lower left image (×400). C, SgII mRNA levels were measured in fractions of cardiomyocytes (n = 5), endothelial cells (n = 2), and non-cardiomyocytes, non-endothelial cells (n = 5) extracted from LV tissue. Gene expression was measured by RT-qPCR and is presented as fold change ± SEM vs. levels in the cardiomyocyte fraction. **p<0.001, *p<0.01.

In parallel with observed alterations in SgII mRNA expression, protein levels of SgII were significantly increased in both the non-infarcted (35% increase, p = 0.007) and the infarcted region of the LV (85% increase, p<0.001) in HF animals as measured by radioimmunoassay (RIA) (Figure 2A). As previously reported for chromogranins [6], [17], SgII immunoreactivity was evident in cardiomyocytes, however, we also found SgII immunoreactivity in endothelial cells, fibroblasts, and other infiltrative cells of the border zone and the infarcted area (Figure 2B). The production of SgII in several cell types of the myocardium was validated by demonstrating similar SgII mRNA levels in fractions of cardiomyocytes, endothelial cells, and non-cardiomyocyte, non-endothelial cells (Figure 2C).

**Figure 3:**
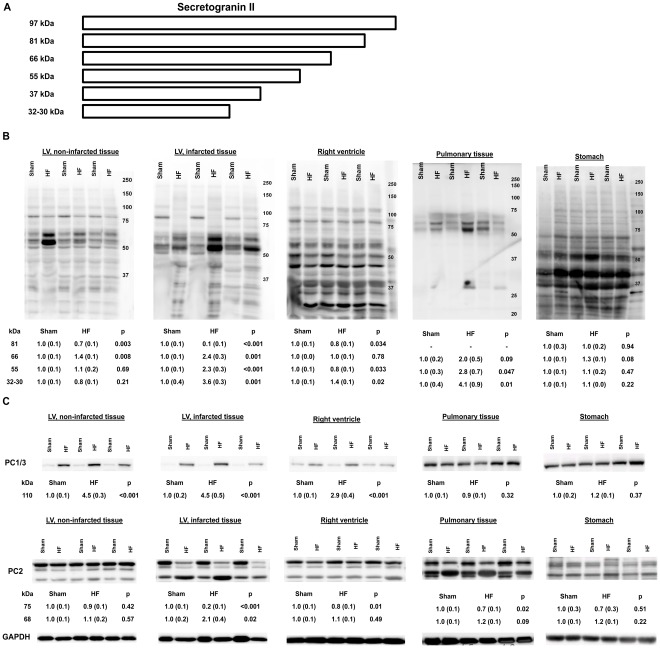
SgII processing is increased in the myocardium of HF mice. A, Figure of SgII processing as reported in non-cardiac tissue (modified from ref. 18). B, SgII processing in myocardial tissue, pulmonary tissue, and tissue from the stomach. Bands at 81, 66, 55, and 32-30 kDa were measured and are presented as fold change (SEM) vs. sham animals (n = 6 for each group). C, Levels of the proteases PC1/3 and PC2 in HF and sham animals.

The complex processing of SgII (Figure 3A) [3], [18] was examined by immunoblotting, and we identified increased processing of SgII to shorter SgII fragments in the myocardium of HF animals compared to sham animals (Figure 3B). Finally, levels of the SgII protease PC1/3 were potently increased in both the left and right ventricle of HF animals, while the active form of the SgII protease PC2 (68 kDa) [19] was only increased in infarcted LV tissue of HF animals (110% increase, p = 0.02) (Figure 3C).

### SgII Production was not Increased in Other Tissues Investigated

**Figure 4:**
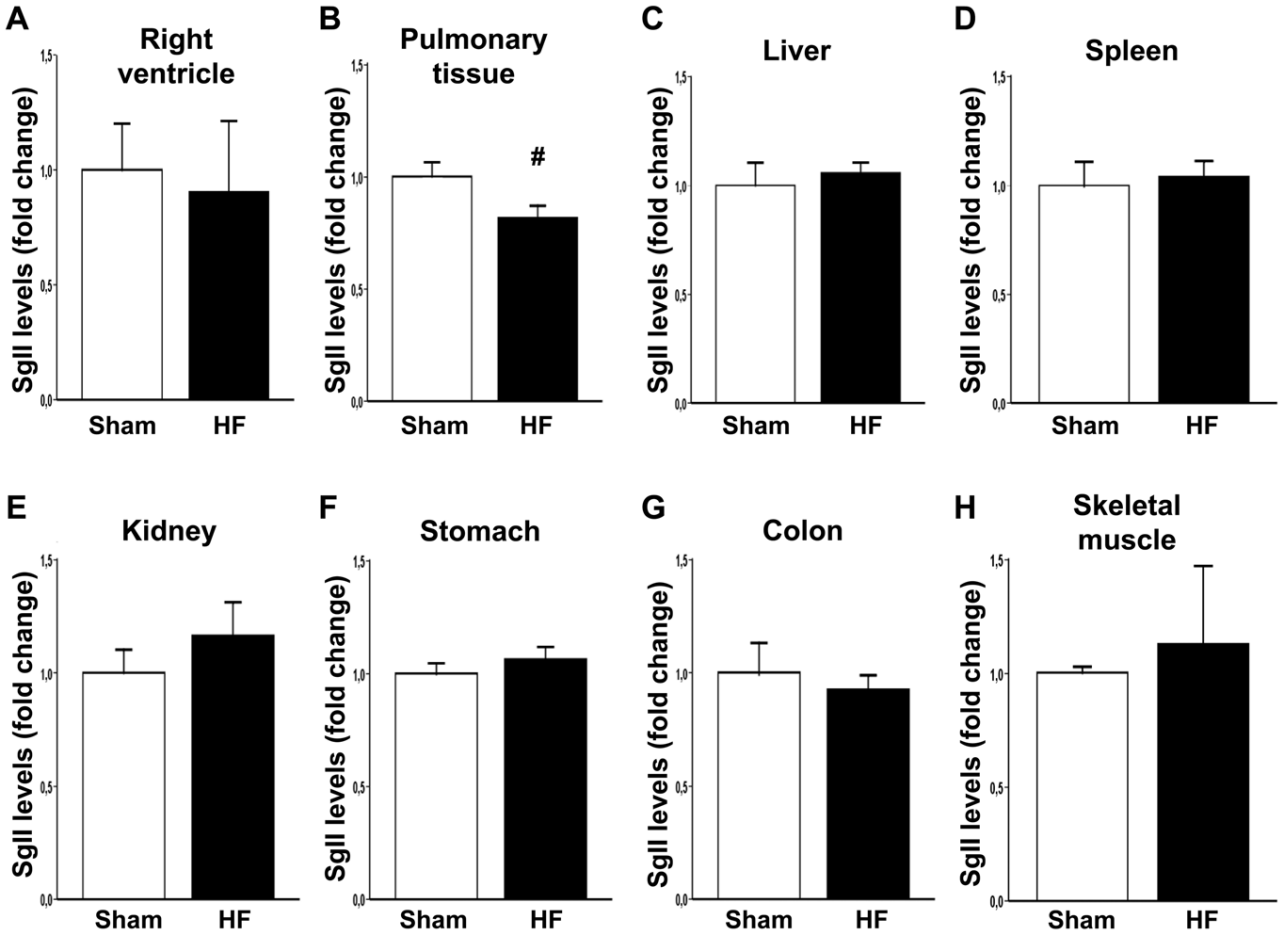
SgII production outside of the left ventricle in heart failure. SgII levels were decreased in pulmonary tissue during HF development, while levels were unchanged in the other tissues examined. SgII levels in the (A) right ventricle, (B) pulmonary tissue, (C) liver, (D) spleen, (E) kidney, (F) stomach, (G) colon, and (H) skeletal muscle were measured by RIA and are presented as fold change ± SEM (n = 6 for both groups, except pulmonary tissue: HF: n = 14, sham: n = 13). # p<0.05.

To further study SgII production in HF, we measured SgII protein levels in non-cardiac tissue by RIA. As illustrated in Figure 4, SgII levels were not altered in the right ventricle, liver, spleen, kidney, stomach, colon, and skeletal muscle during HF development, while SgII levels were decreased by 19% (p = 0.03) in pulmonary tissue of HF animals. The immunoreactivity of short SgII bands was stronger in pulmonary tissue of HF animals (Figure 3B) although PC1/3 or PC2 activity were not significantly increased (Figure 3C). We did not see changes in SgII processing in the other organs examined (immunoblot of tissue from the stomach is included as an example in Figure 3).

### Circulating SgII Levels are Increased in HF Mice

Circulating SgII levels were increased in HF animals (n = 14) compared to sham animals (n = 8): 0.44±0.04 vs. 0.35±0.01 nmol/L, p = 0.049. In the HF group, animals with SgII levels above the mean had more pronounced LV dilatation in systole compared to the other HF mice: 5.7±0.1 vs. 5.4±0.1 mm, p = 0.04.

### Transforming Growth Factor-β and Norepinehrine Increase SgII Gene Expression in Cardiomyocytes

**Figure 5:**
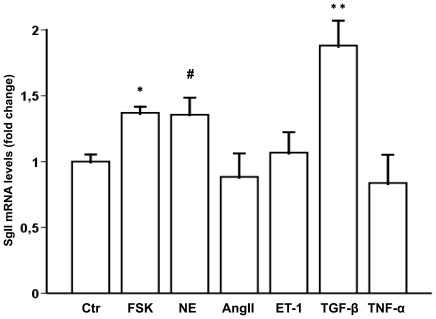
Regulation of cardiomyocyte SgII expression by important hormonal and paracrine factors in HF. SgII mRNA levels were measured by RT-qPCR after stimulating neonatal rat cardiomyocytes for 24 h with either PBS (Ctr, n = 9), forskolin (FSK n = 5), norepinephrine (NE, n = 5), angiotensin II (AngII, n = 4), endothelin-1 (ET-1, n = 5), transforming growth factor-β (TGF-β, n = 6), or tumor necrosis factor-α (TNF-α, n = 6). SgII mRNA levels are presented as fold change ± SEM vs. PBS-stimulated cells. **p<0.001, *p<0.01, # p<0.05.

As multiple endocrine and paracrine factors are known to influence cardiomyocyte protein synthesis [1], we examined possible factors that could influence SgII production in cardiomyocytes. Stimulating neonatal rat cardiomyocytes *in vitro* for 24 h with transforming growth factor-β (TGF-β) and norepinephrine (NE) increased SgII mRNA levels by 85% (p<0.001) and 35% (p = 0.02), respectively, while exposure to angiotensin II (AngII), endothelin-1 (ET-1), and tumor necrosis factor-α (TNF-α) did not affect SgII production (Figure 5). Forskolin (FSK), which has been shown to induce SgII mRNA expression in other cell types [20], was used as a positive control and increased SgII mRNA levels in isolated cardiomyocytes by 35% (p = 0.001).

### The SgII Fragment Secretoneurin Reduces Ischemia/Reperfusion Injury in the Isolated Perfused Rat Heart

**Figure 6:**
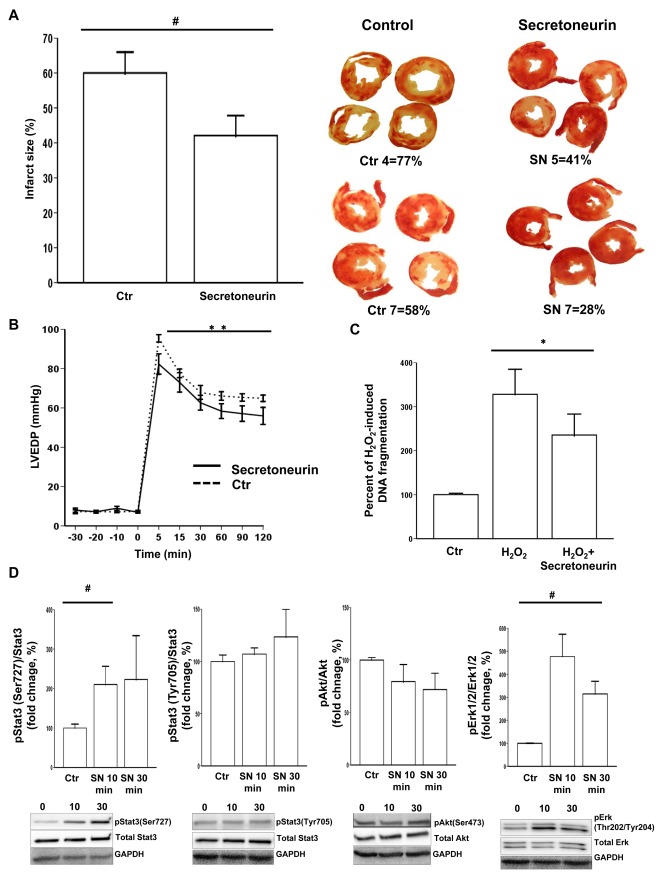
The secretogranin II fragment secretoneurin has protective effects during myocardial ischemia and cardiomyocyte stress. A, Secretoneurin (SN) perfusion reduces infarct size by 30% (upper left) as demonstrated in representative TTC stained images (upper right) after global ischemia in the isolated perfused rat heart. B, Secretoneurin perfusion also improves myocardial function as assessed by LV end-diastolic pressure (LVEDP) after I/R injury. C, Cardiomyocyte apoptosis *in vitro* after H_2_O_2_ exposure was attenuated by secretoneurin stimulation. Cells were extracted from 5 different cell isolations (n = 5 for all groups). D, Short-term stimulation of cardiomyocytes with 10 µg/mL secretoneurin activated protective intracellular pathways as reflected by increased Stat3 and Erk1/2 phosphorylation (n = 5 for all groups). **p<0.001, *p<0.01, # p<0.05.

As SgII production was increased in the infarcted LV of post-infarction HF mice, and infarct size is a major determinant for HF development [21], we assessed whether the SgII fragment secretoneurin could protect against damage after myocardial ischemia. In a global ischemia/reperfusion (I/R) model of the isolated perfused rat heart, the addition of secretoneurin to the buffer reduced infarct size by 30% (p = 0.047) after 30 min of ischemia and 2 h of reperfusion (Figure 6A). The beneficial effects of secretoneurin on I/R damage were also evidenced by lower LV end-diastolic pressure in the hearts perfused with secretoneurin (Figure 6B).

### Secretoneurin Stimulation Increases Stat3 and Erk1/2 Phosphorylation and Reduces Hydrogen Peroxide-induced Apoptosis in Isolated Cardiomyocytes

Reduced apoptosis in cardiomyocytes at risk could be a mechanism by which secretoneurin protects the myocardium after I/R injury. We therefore investigated whether secretoneurin plays a role in the protection of cardiomyocytes exposed to hydrogen peroxide (H_2_O_2_). Exposure of H_2_O_2_ to cardiomyocytes markedly induced apoptosis, as indicated by measurements of DNA fragmentation (Figure 6C). We observed that the proportion of apoptotic cells after H_2_O_2_ exposure was 30% lower (p = 0.005) in cells co-stimulated with secretoneurin compared to cells in standard medium. Finally, we examined signaling pathways that could account for the effects of secretoneurin on I/R injury and cardiomyocyte apoptosis. Phospho Akt_Ser473_, Erk1/2_Thr202/Tyr204_, and Stat3_Ser727/Tyr705_ levels were measured in cardiomyocytes after short-term secretoneurin exposure. Secretoneurin stimulation significantly increased Erk1/2_Thr202/Tyr204_ phosphorylation (four and two fold increase after 10 and 30 min, respectively, p = 0.04) (Figure 6D). Stat3_Ser727_ phosphorylation was increased after 10 min of secretoneurin exposure (100% increase, p = 0.04), while phospho Stat3_Tyr705_ and Akt_Ser473_ levels were unaltered.

### Circulating Levels of SgII are Increased in Patients with Chronic, Stable HF

**Figure 7:**
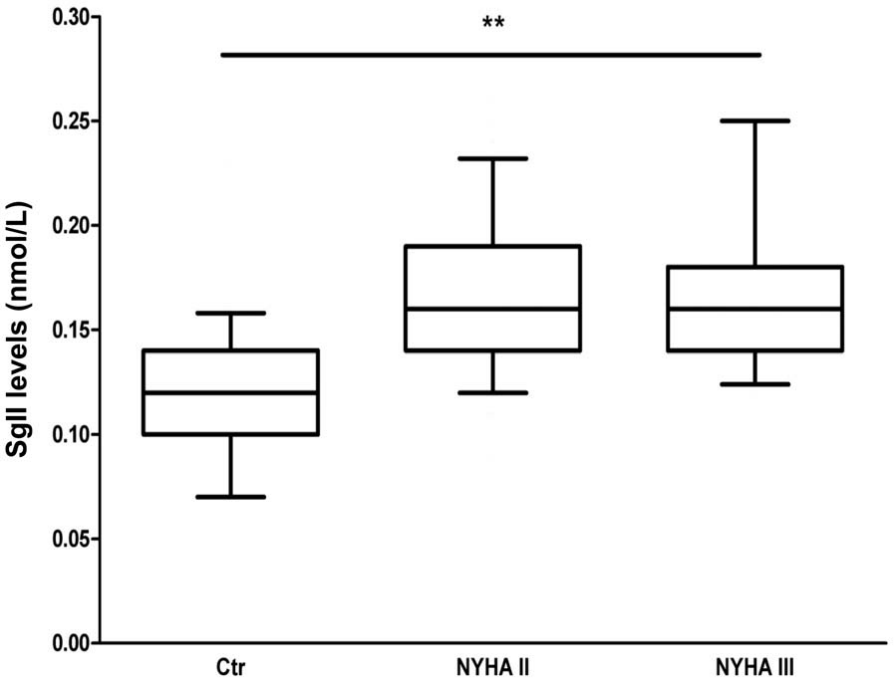
Circulating SgII levels are elevated in patients with chronic, stable HF. SgII levels were significantly increased in HF patients (n = 58) compared to healthy age- and gender-matched control subjects (n = 20): Median 0.16 (Q1–3 0.14–0.18) vs. 0.12 (0.10–0.14) nmol/L, p<0.001. HF patients are presented according to NYHA functional class. The horizontal line within the box represents the median level, the boundaries of the box the 25^th^ and 75^th^ percentile levels, and the whiskers the 10^th^–90^th^ percentile. **p<0.001.

To validate the results of the experimental model, and to demonstrate increased SgII levels also in chronic, stable HF, circulating SgII levels in 58 patients with chronic HF were compared to levels in 20 age- and gender-matched healthy control subjects (Table 2). In this cohort of stable HF patients treated according to updated guidelines, SgII levels were higher than levels in the control group: median 0.16 (Q1–3 0.14–0.18) vs. 0.12 (0.10–0.14) nmol/L, p<0.001 (Figure 7). Plasma levels of SgII correlated with BNP in HF patients (r = 0.26, p = 0.05), but not in control subjects (r = 0.19, p = 0.43). SgII levels did not correlate with CgA or CgB levels in control subjects or HF patients (Supporting Information Table S2). As evaluated by receiver operating analysis, circulating levels of SgII were more closely regulated in HF than both CgA (AUC = 0.84 for SgII vs. AUC = 0.57 for CgA, p = 0.001) and CgB levels (AUC = 0.68 for CgB, p = 0.03 vs. SgII). Use of a proton pump inhibitor (PPI), a medication known to increase circulating CgA levels [6], did not increase SgII levels as levels in patients using PPI (n = 8) were similar to SgII levels in the other HF patients: 0.16 (Q1–3 0.13–0.19) vs. 0.16 (0.14–0.18) nmol/L, respectively, p = 0.68).

**Table 2:** Descriptive statistics of heart failure patients and control subjects.

	Control subjects(n = 20)	HF patients(n = 58)	p
Male sex (n, %)	16 (80%)	47 (81%)	0.92
Age, years (mean ± SEM)	60.6±1.1	62.8±1.6	0.43
NYHA class (n, %)			
II		47 (81%)	
III		11 (19%)	
Etiology for HF (n, %)			
Ischemic		35 (60%)	
Dilated cardiomyopathy		21 (36%)	
Other		2 (4%)	
Duration of HF, months (median, Q1–3)		18 (12–48)	
LVEF, % (mean ± SEM)		33±1	
Medication (n, %)			
β-blocker		57 (98%)	
ACEI		41 (71%)	
ARB		17 (29%)	
ACEI or ARB		58 (100%)	
Aldosterone antagonist		11 (19%)	
Diuretic		43 (74%)	
Statin		38 (65%)	
Warfarin		29 (50%)	
ASA		34 (59%)	
Clopidogrel		7 (12%)	
Digitalis		14 (24%)	
Amiodarone		6 (10%)	
Nitrate		6 (10%)	
PPI		8 (14%)	
CRT		10 (17%)	
ICD		11 (19%)	
CgA levels, nmol/L	4.5 (4.0–5.3)	5.0 (3.5–8.2)	0.33
CgB levels, nmol/L	1.47 (1.39–1.58)	1.63 (1.44–1.80)	0.02
BNP levels, pg/mL	26 (13–37)	197 (89–338)	<0.001

NYHA class indicates New York Heart Association functional class; Q1–3, quartile 1–3; LVEF, left ventricular ejection fraction; ACEI, angiotensin converting enzyme inhibitor; ARB, angiotensin II receptor blocker; ASA, acetyl salicylic acid; PPI, proton pump inhibitor; CRT, cardiac resynchronization therapy; ICD, implantable cardioverter-defibrillator; CgA, chromogranin A; CgB, chromogranin B; and BNP, B-type natriuretic peptide. Biomarker levels are presented as median (quartile 1–3).

## Discussion

In the present study we demonstrate for the first time that the expression of SgII, a member of the granin protein family, is increased in the LV during HF development. Moreover, SgII processing to shorter fragments is increased in the myocardium in post-infarction HF, which could be beneficial as the SgII fragment secretoneurin protects against myocardial I/R injury and cardiomyocyte apoptosis. SgII production was not increased in other tissues investigated, and therefore the heart could be a significant contributor to the elevated circulating SgII levels in patients with HF.

### Myocardial Production of SgII in HF

HF is associated with increased myocardial production of several protein families [1], [2]. Like other granin proteins, SgII is a pro-hormone with multiple dibasic cleavage sites [3]. Although the processing of SgII is known to be more complex than for most other pro-hormones [3], [22], SgII has in several tissues been demonstrated to be almost fully processed to the short 33 amino acid peptide secretoneurin [23]. In the circulation, the majority of SgII immunoractivity is also reportedly found in the form of secretoneurin [23]. Pertinent to this, data from non-cardiac tissue have indicated that cells with an enhanced secretory rate exhibit increased processing of SgII to shorter fragments [18]. Accordingly, our novel data demonstrating increased levels and processing of SgII to shorter fragments in the myocardium during post-myocardial infarction HF point to the heart as a significant contributor to the increased circulating levels of SgII in HF. This is supported by the lack of increase in SgII production in non-cardiac tissue in HF as measured by RIA. Still, as immunoreactivity of short SgII bands in the lungs of HF animals were increased, we cannot preclude a contribution also by pulmonary tissue to circulating SgII levels. However, the full-length pro-form of SgII was not evident in pulmonary tissue and no increment in PC1/3 or PC2 activity was demonstrated. Thus, another possibility is leakage of SgII immunoreactivity from the circulation to the lungs due to severe pulmonary congestion. Mass spectrometry peptide mass fingerprinting of a very strong band in Commassie stained gels from pulmonary tissue of HF mice identified albumin, which is a testament to the pronounced congestion and leakage of proteins into the lungs of our HF mice.

The mechanism by which SgII processing is increased seems to be enhanced PC1/3 (previously denoted PC1 or PC3) and PC2 activity in the LV, which are the principal proteases of SgII [15], [16]. The complex processing of SgII in the myocardium with several shorter fragments corresponds to immunoblots of SgII processing in non-cardiac tissue [15], [16], [18], [22], [23]. One report has previously demonstrated increased PC1/3 mRNA levels close to the infarct area in rats with myocardial infarction and HF [24], but to our knowledge the increase in both PC1/3 and PC2 in the LV of animals with HF has previously not been reported. Increased PC1/3 and PC2 activity has been hypothesized as a conceptual model for enhanced SgII processing in non-cardiac cells [18].

We identified cardiomyocytes as contributors to the SgII production in the myocardium. This is in line with previous data for chromogranins [6], [17], and with a report published during progression of our work that demonstrates the presence of SgII in the healthy rodent myocardium [25]. We have also previously demonstrated that NE, TGF-β, and AngII regulate CgB production in cardiomyocytes [6]. Similar to this, we now report that TGF-β and NE both induce SgII mRNA expression in cardiomyocytes *in vitro*, while AngII failed to increase SgII mRNA levels. Distinct regulation of different granins has been reported in other cell types and may be explained by variations in the promoter region, including the serum response element (SRE), which is only found in the SgII promoter [20]. Effect of growth factors on SRE promoter sites could explain the potency of TGF-β as a stimulus for SgII production in cardiomyocytes [20], but the precise mechanism regulating cardiomyocyte SgII production needs to be established in future studies. This also relates to a possible role of hypoxia-inducible factor-1α (HIF-1α) as a regulator of SgII production after myocardial ischemia. Indeed, HIF-1α has previously been found to increase SgII production in skeletal muscle subjected to hypoxia [12], and we found SgII production increased in the LV of animals with myocardial infarction and HF. However, in contrast to data for chromogranins [6], [17], we also find SgII to be produced by non-cardiomyocyte cardiac cells, including endothelial cells and cell fractions with a high proportion of cardiac fibroblasts. The mechanism controlling SgII production in non-cardiomyocytes needs to be established in subsequent studies, along with the relative contribution by the different cell types to myocardial SgII production in HF.

### Functional Aspects of the SgII Fragment Secretoneurin in the Myocardium

Proteins produced by the heart during the development of HF may exert their actions via endocrine, paracrine or autocrine mechanisms [1]. Findings in the current study suggest that SgII and the fragment secretoneurin could exert important effects in the myocardium by reducing I/R injury and cardiomyocyte apoptosis. These effects of secretoneurin, together with the observation that LV SgII levels and processing are increased in animals with myocardial infarction and HF, are compatible with known functional properties of secretoneurin. Secretoneurin has previously been found to attract leukocytes, especially monocytes [26], and may be of importance for the early post-infarction inflammatory response in the myocardium. Secretoneurin also attracts endothelial cells, both mature endothelial cells for angiogenesis [27] and bone marrow-derived progenitor endothelial cells for vasculogenesis [28], suggesting that secretoneurin could play a role in revascularization of infarcted myocardial tissue. Hence, the mechanism by which secretoneurin induces revascularization are likely both recruitment of bone-marrow derived cells to produce new vessels and local sprouting of vessels [13], [14], potentially mediated by a nitric oxide-dependent mechanism analogous to what has been reported in the hindlimb ischemia model [14].

In this study, we have shown for the first time that secretoneurin attenuates cardiomyocyte apoptosis and reduces infarct size in the subacute phase after myocardial infarction. A putative mechanism for this effect of secretoneurin against I/R injury is via reduction of cardiomyocyte apoptosis in the border zone, possibly mediated by Erk1/2 and Stat3 activation [29], [30]. Stat3 may represent a direct link between secretoneurin and reduced opening of the mitochondrial permeability transition pore [31], whereas the mechanism by which Erk1/2 reduces apoptosis seems to be mediated through additional downstream molecules [32]. Other studies have also shown secretoneurin to protect endothelial cells from apoptosis through Erk1/2 signaling [27], [28], and to attenuate neuronal cell death after ischemia by induction of the Jak/Stat pathway [13], providing support for secretoneurin as a protective factor in the subacute phase of tissue ischemia. Hence, based on our data of increased SgII production and processing in the infarcted area, SgII and secretoneurin production seem to represent an endogenous protective mechanism during post-infarction HF development. With infarct size being considered a major determinant of post-infarction HF development [21], we believe these results are relevant also for myocardial function in the more chronic stages of HF. However, we have not assessed the long-term effects of increased SgII levels for organ function in HF and this should be explored in future studies. Before the long-term effects of secretoneurin have been established, the net effect by increased SgII levels in HF cannot be estimated, although we provide evidence that LV SgII production seems to be protective in the subacute phase of post-infarction HF development.

The exact mechanism by which secretoneurin mediates the protection during I/R injury and cardiomyocyte apoptosis is yet to be established. A previous report suggested that monocytes have functionally active binding sites for secretoneurin [33], but after more than 25 years of research, the putative receptor for secretoneurin has not been characterized. The lack of information on the secretoneurin receptor and the intracellular signaling pathways induced by secretoneurin are considered the main obstacle preventing a more detailed understanding of SgII in health and disease [3]. However, based on our work and the work of other groups [13], [27], [28], Stat3 and Erk1/2 signaling seem to be of paramount importance for the protection by secretoneurin during I/R injury and apoptosis.

### Circulating SgII Levels in Patients with HF

In this first report on SgII in HF, we find circulating SgII levels to be increased in the early post-infarction phase of mice with HF and in patients with chronic, stable HF. The modest correlation between SgII and BNP levels suggests different pathophysiology behind SgII and BNP production and release in HF. This was also evident by the lack of correlation between LV SgII and BNP mRNA levels in our HF mice. Accordingly, SgII levels seem to represent specific pathophysiology in HF not covered by BNP.

The potential of SgII as a new HF biomarker should be assessed in relation to the increasing recognition of the other granin proteins CgA and CgB as candidate HF biomarkers [2], [34]. CgA has been demonstrated to provide incremental prognostic information to established indices of risk, including BNP, in several cohorts of patients with cardiac disease [4], [7]–[9], while CgB has been proposed as a marker associated with cardiomyocyte calcium handling [35]. Accordingly, that we find circulating SgII levels to better discriminate HF patients from control subjects compared to CgA and CgB levels indicates a potential also for SgII as a new HF biomarker. Whether high SgII levels in patients with stable HF are primarily reflective of I/R injury and cardiomyocyte apoptosis is not clear, but given the multifaceted properties of secretoneurin and SgII [3], additional pathophysiology may be associated with SgII as a biomarker and this should be explored in future studies.

### Conclusion

We have observed increased myocardial SgII production and processing to shorter fragments in animals with myocardial infarction and HF. This could be beneficial as the SgII fragment secretoneurin protects from I/R injury and cardiomyocyte apoptosis. As SgII production was not enhanced in other tissues investigated, LV SgII production could be a significant contributor to the elevated circulating levels of SgII in patients with HF.

## Methods

Animal experiments were performed according to recommendations from the European Council for Laboratory Animal Science and approved by the Norwegian Council for Animal Research (#HR0506). The study protocol of the clinical study was approved by the South-Eastern Regional Ethics Committee Norway (#1.2006.2653) before the initiation of the study, and all participants gave their written informed consent prior to study commencement.

### Mouse Model of HF

Mice were anesthetized with 0.2 mg propofol in the tail vein, trachetomized, connected to an animal ventilator, and ventilated with a mixture of 98% oxygen and 2% isoflurane during surgery. A permanent ligation of the left main coronary artery was performed in mice and the animals were evaluated by echocardiography for development of HF [36]. The echocardiographic study was performed in 2-D view and per standard guidelines [37] by Vivid 7 (GE Vingmed, Horten, Norway). Images were stored in Echopac (GE Vingmed) and later processed and evaluated by an experienced researcher in small animal echocardiography (IS) with no knowledge of circulating SgII levels. Sham-operated (sham) animals underwent the same procedure without coronary artery ligation. All efforts were made to minimize suffering. Animals were sacrificed at one week post-surgery, when hearts and other organs were dissected, prepared, and stored as previously described [6].

### RT-qPCR, Radioimmunoassays, Immunhistochemistry, and Immunoblotting

mRNA levels were measured with TaqMan Gene Expression assays from Applied Biosystems (Foster City, CA, USA): (i) mouse myocardium: SgII (Mm00843883_s1), CgA (Mm00514341_m1), CgB (Mm00483287_m1), BNP (Mm00435304_g1), ribosomal protein L4 (RPL4) (Mm00834993_g1); and (ii) neonatal rat cardiomyocytes: SgII (Rn01400686_g1) and RPL4 (Rn00821091_g1). SgII protein levels in tissue and plasma were measured by a RIA binding to the secretoneurin region of SgII (SgII_154–165_) [38]. The detection limit of the SgII RIA in plasma is 0.05 nmol/L and the assay has a coefficient of variation (CV) of 9% in the lower range (1.10 nmol/L) and 4% in the upper range (3.80 nmol/L). We used a commercial RIA for CgA analysis (EuroDiagnostica AB, Malmö, Sweden) with a detection limit of 0.8 nmol/L and a CV of 13% in the lower range (3.1 nmol/L) and 9% in the upper range (17.0 nmol/L), while an in-house RIA was used for CgB analysis with detection limit ≥0.80 nmol/L and a CV of 17% in the lower range (1.40 nmol/L) and 8% in the upper range (6.40 nmol/L) [39]. For immunoblotting we used an N-terminal SgII antibody (ab20246, Abcam, Cambridge, UK) as SgII processing has been reported to start from the C-terminal end [18]. We quantitated four bands of SgII (81, 66, 55, and 32-30 kDa) according to previous data on SgII processing in non-cardiac tissue [18]. Additional antibodies for immunoblotting and immunhistochemistry are reported in Supporting Information S2.

### Regulation of SgII mRNA Production in Neonatal Rat Cardiomyocytes

After 24 h starvation of the cells, neonatal rat cardiomyocytes were stimulated as previously reported [6] for 24 h with the following agents; forskolin (FSK) [10 µM], norepinephrine (NE) [100 µM], endothelin-1 (ET-1) [250 ng/mL], angiotensin II (AngII) [1 µM] (all Sigma-Aldrich, St. Louis, MO, USA), tumor necrosis factor-α (TNF-α) [10 ng/mL] (BioSource International, Camarillo, CA, USA), and transforming growth factor-β (TGF-β) [10 ng/mL] (R&D Systems, Minneapolis, MN, USA).

### Langendorff Perfusion

All rats were anesthetized with 5% sodium pentobarbital (60–80 mg/kg intraperitoneally (i.p)) and heparinised (500 IU i.p). After anesthesia, rat hearts were rapidly excised and placed in ice-cold Krebs-Henseleit Buffer (KHB) (mmol/L: NaCl 118.5; NaHCO_3_ 25; KCl 4.7; KH_2_PO_4_ 1.2; MgSO_4_/7H_2_O 1.2; glucose/1H_2_O 11.1; CaCl_2_ 1.8) for further dissection. After aortic cannulation, the hearts were mounted on a Langendorff system (AD Instruments Pty Ltd, Castle Hill, NSW 2154, Australia) and retrogradely perfused with warm (37°C), oxygenated (95% O_2_, 5% CO_2_) KHB at constant pressure of 70 mmHg. After 40 min of stabilization, hearts were subjected to 30 min of global ischemia, and then reperfused for 120 min (Supporting Information Figure S1). In the experimental group (secretoneurin group), 0.66 µg/mL secretoneurin (NeoMPS, Strasbourg, France) was added to the perfusate 20 min prior to ischemia, and was also used throughout the reperfusion period. The effect of local production was taken in account when deciding the concentration of secretoneurin for the Langendorff model as previous reports on other secreted proteins have demonstrated that local tissue concentrations and levels in supernatants of stimulated cells may be >100 fold higher than levels observed in the peripheral circulation [40], [41]. A recirculation system was inserted from 20 min prior to induction of ischemia in both groups (Supporting Information Figure S1), and used throughout the reperfusion period. Infarct size was calculated with Adobe Photoshop (Adobe Systems, San Jose, CA, USA) as percentage of the total area by an investigator blinded to treatment.

### Effect of Secretoneurin on Phosphoproteins

To assess the short-term effect of secretoneurin stimulation on phosphoproteins, the cardiomyocytes were stimulated for 10 or 30 min with 10 µg/mL secretoneurin (NeoMPS) or vehicle. Cells were harvested in lysis buffer (Tris pH 7.6, 5 M NaCl, 0.5 M EDTA, 0.1 M EGTA, 1 M B-gly [Sigma-Aldrich St. Louis, MO, USA] and NP-40), and the protein levels measured by immunoblotting (details reported in Supporting Information S2).

### Cell Death Enzyme-linked Immunosorbent Assay

Neonatal rat cardiomyocytes were stimulated with 10 µg/mL secretoneurin (NeoMPS) or vehicle for 24 h, followed by 24 h exposure to hydrogen peroxide (H_2_O_2_, 100 µmol) in the presence of secretoneurin or vehicle. Cells were then harvested in lysis buffer and apoptosis assessed using the Cell Death Enzyme-linked Immunosorbent Assay (Cell Death Detection ELISA^PLUS^, Roche, Basel, Switzerland) according to manufacturer procedure.

### Patients with Stable HF and Control Subjects

Fifty-eight patients with echocardiographically documented impaired systolic function [LV ejection fraction (LVEF) ≤50%], and no hospitalization for HF during the last three months prior to study commencement, were compared to 20 control subjects with no history or current symptoms of chronic disease. The control subjects had a normal physical examination, did not use medication regularly, and were hospital employees or recruited from outside of the hospital.

### Statistical Analysis

Continuous data are presented as mean (± SEM) or median (quartile [Q] 1–3) and categorical values as counts (percentage). Continuous variables were compared by Student’s *t*-test except circulating biomarker values in patients that were compared by the Mann-Whitney U-test due to a severe right-skewed distribution (p<0.001). Serial data were compared by Two-Way ANOVA, and categorical data by the chi-square test or Fisher’s exact test as appropriate. Correlations were calculated by Spearman rank correlation. P-values <0.05 were considered significant for all analyses. Statistical analyses were performed with SPSS for Windows version 16.0 (SPSS, Chicago, IL) except receiver operating analysis which was performed with MedCalc for Windows, version 9.5.1.0 (MedCalc Software, Mariakerke, Belgium) by the method of Hanley and McNeil [42].

Details regarding methods can be found in the Supporting Information S2.

## Supporting Information

Figure S1 Hearts from male adult rats were rapidly excised and mounted on a Langendorff system. After 40 min of stabilization, hearts were subjected to 30 min of global ischemia, and then reperfused for 120 min. In the experimental group (secretoneurin group), 0.66 µg/mL secretoneurin (SN) was added to the perfusate 20 min prior to ischemia, and also used throughout the reperfusion period.
(TIF)

Table S1 Correlations between mRNA levels of granins and BNP in the left ventricle of heart failure and sham-operated mice.
(DOC)

Table S2 Correlations between circulating levels of granin proteins and BNP in patients with heart failure and healthy control subjects.
(DOC)

Supporting Information S1 Enhanced albumin levels in pulmonary tissue of HF animals were identified by mass spectrometry peptide mass fingerprinting. Coomassie stained protein bands were cut out from SDS-polyacrylamide gels and identified by mass spectrometry peptide mass fingerprinting. A, Representative Commassie stained gel from pulmonary tissue with arrow indicating extracted band for mass spectrometry peptide mass fingerprinting. B, Results for the mass spectrometry peptide mass fingerprinting identifying albumin for all 8 samples after searching the NCBI database. Significant hits for sample 1.1 are given below and they all represent slightly different variants of albumin. The same proteins were reported for the other 7 samples. All the main signals in the spectra are assigned to albumin.
(DOC)

Supporting Information S2 Supplemental methods.
(DOC)
