# Supplementary figures and images for: Secretogranin II; a Protein Increased in the Myocardium and Circulation in Heart Failure with Cardioprotective Properties
Source: PLoS One. 2012 May 24;7(5):e37401. doi: 10.1371/journal.pone.0037401 (PMC3360055; doi:10.1371/journal.pone.0037401)

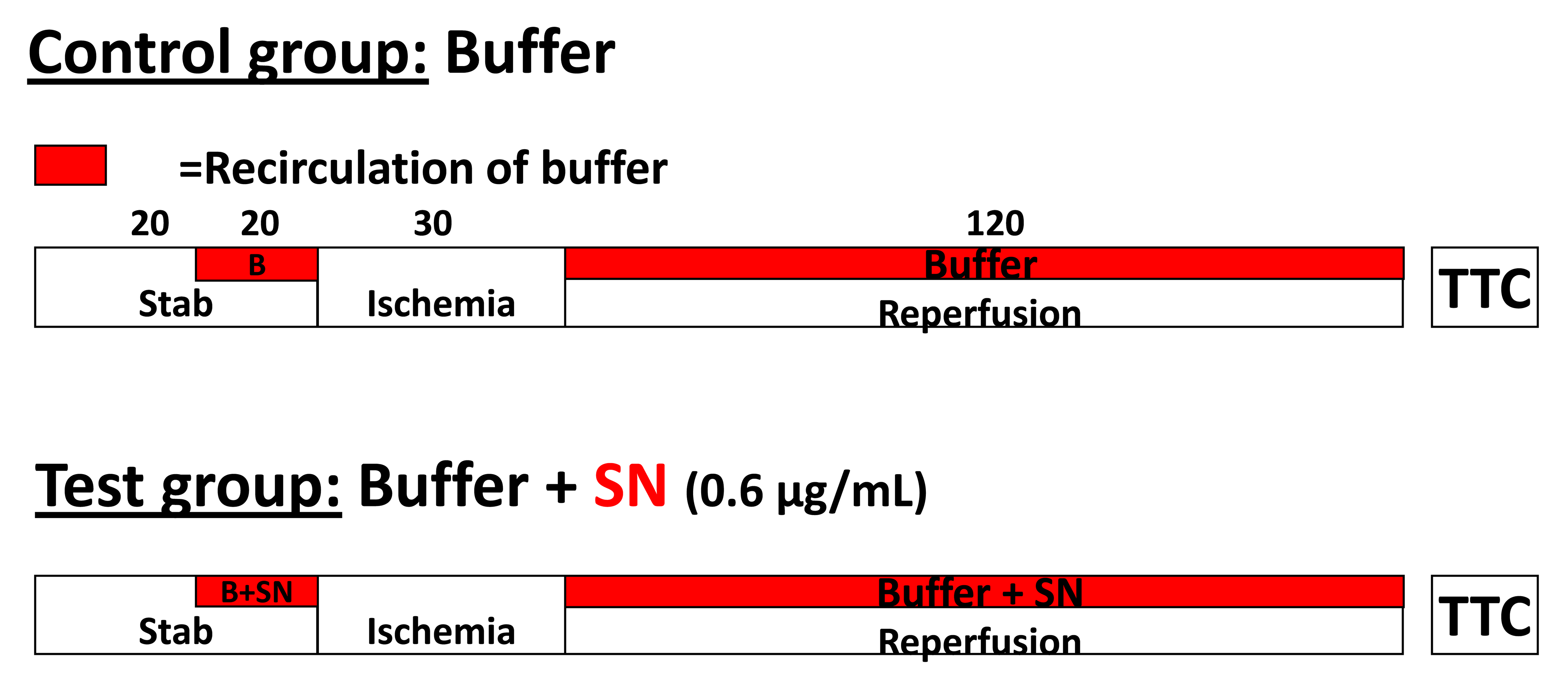

Supplement: Figure S1 — Hearts from male adult rats were rapidly excised and mounted on a Langendorff system. After 40 min of stabilization, hearts were subjected to 30 min of global ischemia, and then reperfused for 120 min. In the experimental group (secretoneurin group), 0.66 µg/mL secretoneurin (SN) was added to the perfusate 20 min prior to ischemia, and also used throughout the reperfusion period. (TIF) [file pone.0037401.s001.tif]
