# Supplementary material for: Secretogranin II; a Protein Increased in the Myocardium and Circulation in Heart Failure with Cardioprotective Properties
Source: PLoS One. 2012 May 24;7(5):e37401. doi: 10.1371/journal.pone.0037401 (PMC3360055; doi:10.1371/journal.pone.0037401)
Supplement: Table S1 — Correlations between mRNA levels of granins and BNP in the left ventricle of heart failure and sham-operated mice. (DOC) [file pone.0037401.s002.doc]

|  | **SgII mRNA levels** | |
| --- | --- | --- |
|  | **Sham animals** | **HF animals** |
| **CgA mRNA levels** | r= 0.81, p= 0.02 | r= 0.68, p= 0.04 |
| **CgB mRNA levels** | r= 0.10, p= 0.82 | r= 0.18, p= 0.64 |
| **BNP mRNA levels** | r= -0.02, p= 0.96 | r= 0.38, p= 0.31 |
