# Supplementary material for: Secretogranin II; a Protein Increased in the Myocardium and Circulation in Heart Failure with Cardioprotective Properties
Source: PLoS One. 2012 May 24;7(5):e37401. doi: 10.1371/journal.pone.0037401 (PMC3360055; doi:10.1371/journal.pone.0037401)
Supplement: Table S2 — Correlations between circulating levels of granin proteins and BNP in patients with heart failure and healthy control subjects. (DOC) [file pone.0037401.s003.doc]

|  | **SgII levels** | |
| --- | --- | --- |
|  | **Control subjects** | **HF patients** |
| **CgA levels** | r= -0.26, p= 0.27 | r= 0.16, p= 0.25 |
| **CgB levels** | r= -0.34, p= 0.14 | r= 0.09, p= 0.49 |
| **BNP levels** | r= 0.19, p= 0.43 | r= 0.26, p= 0.05 |
