# Supplementary material for: Secretogranin II; a Protein Increased in the Myocardium and Circulation in Heart Failure with Cardioprotective Properties
Source: PLoS One. 2012 May 24;7(5):e37401. doi: 10.1371/journal.pone.0037401 (PMC3360055; doi:10.1371/journal.pone.0037401)
Supplement: Supporting Information S1 — Enhanced albumin levels in pulmonary tissue of HF animals were identified by mass spectrometry peptide mass fingerprinting. Coomassie stained protein bands were cut out from SDS-polyacrylamide gels and identified by mass spectrometry peptide mass fingerprinting. A, Representative Commassie stained gel from pulmonary tissue with arrow indicating extracted band for mass spectrometry peptide mass fingerprinting. B, Results for the mass spectrometry peptide mass fingerprinting identifying albumin for all 8 samples after searching the NCBI database. Significant hits for sample 1.1 are given below and they all represent slightly different variants of albumin. The same proteins were reported for the other 7 samples. All the main signals in the spectra are assigned to albumin. (DOC) [file pone.0037401.s004.doc]

**Supporting Information S1**

**to**

**Secretogranin II; a protein increased in the myocardium and circulation in heart failure with cardioprotective properties**

**Helge Røsjø MD^1,2^; Mats Stridsberg MD, PhD^3^; Geir Florholmen MSc, PhD^2,4^; Kåre-Olav Stensløkken MSc, PhD^5^; Anett Hellebø Ottesen MSc^1,2,4^**; **Ivar Sjaastad MD, PhD^2,4^; Cathrine Husberg MSc, PhD^2,4^; Mai Britt Dahl MSc^1,2,6^; Erik Øie MD, PhD^2,7^; William E. Louch MSc, PhD^2,4^; Torbjørn Omland MD, PhD, MPH^1,2^; Geir Christensen MD, PhD, MHA^2,4^**

^1^ Division of Medicine, Akershus University Hospital, Lørenskog, Norway

^2^ Center for Heart Failure Research and K.G. Jebsen Cardiac Research Centre, Institute of Clinical Medicine, University of Oslo, Oslo, Norway

^3^ Department of Medical Sciences, Uppsala University, Uppsala, Sweden

^4^ Institute for Experimental Medical Research, Oslo University Hospital, Ullevål, Oslo, Norway

**^5^** Department of Molecular Biosciences, University of Oslo, Oslo, Norway

^6^ Department of Clinical Molecular Biology, Akershus University Hospital, Lørenskog, Norway

^7^ Research Institute for Internal Medicine, Oslo University Hospital, Rikshospitalet, Oslo, Norway

**A.**


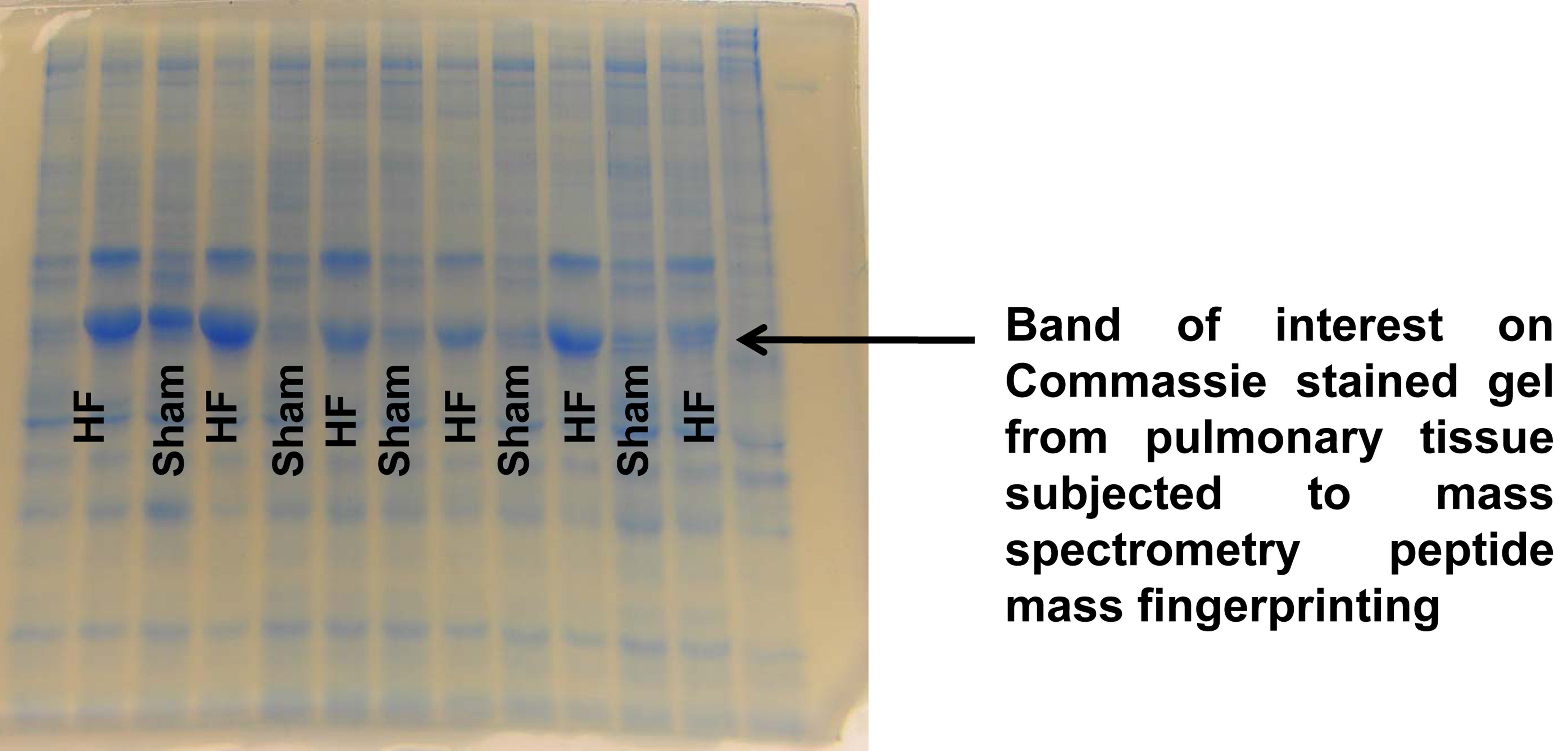


**Sample 1.1**

**Sample 2.2**

**Sample 2.3**

**Sample 2.5**

**Sample 2.4**

**Sample 2.1**

**Sample 2.6**

**Sample 1.6**

**B.**

Significant hits reported for sample 1.1

**albumin [Mus musculus] gi|163310765**

MKWVTFLLLLFVSGSAFSRGVFRREAHKSEIAHRYNDLGEQHFKGLVLIAFSQYLQKCSYDEHAKLVQEVTDFAKTCVADESAANCDKSLHTLFGDKLCAIPNLRENYGELADCCTKQEPERNECFLQHKDDNPSLPPFERPEAEAMCTSFKENPTTFMGHYLHEVARRHPYFYAPELLYYAEQYNEILTQCCAEADKESCLTPKLDGVKEKALVSSVRQRMKCSSMQKFGERAFKAWAVARLSQTFPNADFAEITKLATDLTKVNKECCHGDLLECADDRAELAKYMCENQATISSKLQTCCDKPLLKKAHCLSEVEHDTMPADLPAIAADFVEDQEVCKNYAEAKDVFLGTFLYEYSRRHPDYSVSLLLRLAKKYEATLEKCCAEANPPACYGTVLAEFQPLVEEPKNLVKTNCDLYEKLGEYGFQNAILVRYTQKAPQVSTPTLVEAARNLGRVGTKCCTLPEDQRLPCVEDYLSAILNRVCLLHEKTPVSEHVTKCCSGSLVERRPCFSALTVDETYVPKEFKAETFTFHSDICTLPEKEKQIKKQTALAELVKHKPKATAEQLKTVMDDFAQFLDTCCKAADKDTCFSTEGPNLVTRCKDALA

1 0.070000 Da 0 Trypsin

Carbamidomethyl (C)

136.000000 2 NCBInr gi|163310765

955.528388 0.001090 98 105 0 LCAIPNLR

1298.698174 -0.000099 362 372 0 HPDYSVSLLLR

1438.773061 -0.004924 439 452 0 APQVSTPTLVEAAR

1454.800488 0.001111 361 372 1 RHPDYSVSLLLR

1478.795802 0.007655 422 434 0 LGEYGFQNAILVR

1608.791078 0.008668 348 360 0 DVFLGTFLYEYSR

1661.853909 0.009243 470 483 0 LPCVEDYLSAILNR

1680.838030 0.002138 243 257 0 LSQTFPNADFAEITK

1881.939842 0.010353 509 524 0 RPCFSALTVDETYVPK

1900.903856 0.014848 153 168 0 ENPTTFMGHYLHEVAR

1916.896955 0.013029 153 168 0 ENPTTFMGHYLHEVAR

1980.987743 0.066631 585 602 1 AADKDTCFSTEGPNLVTR

Sequence coverage: 22.9%, MW: 70.7 kDa

**unnamed protein product [Mus musculus] gi|26341396**

NRYNDLGEQHFKGLVLIAFSQYLQKCSYDEHAKLVQEVTDFAKTCVADESAANCDKSLHTLFGDKLCAIPNLRENYGELADCCTKQEPERNECFLQHKDDNPSLPPFERPEAEAMCTSFKENPTTFMGHYLHEVARRHPYFYAPELLYYAEQYNEILTQCCAEADKESCLTPKLDGVKEKALVSSVRQRMKCSSMQKFGERAFKAWAVARLSQTFPNADFAEITKLATDLTKVNKECCHGDLLECADDRAELAKYMCENQATISSKLQTCCDKPLLKKAHCLSEVEHDTMPADLPAIAADFVEDQEVCKNYAEAKDVFLGTFLYEYSRRHPDYSVSLLLRLAKKYEATLEKCCAEANPPACYGTVLAEFQPLVEEPKNLVKTNCDLYEKLGEYGFQNAILVRYTQKAPQVSTPTLVEAARNLGRVGTKCCTLPEDQRLPCVEDYLSAILNRVCLLHEKTPVSEHVTKCCSGSLVERRPCFSALTVDETYVPKEFKAETFTFHSDICTLPEKEKQIKKQTALAELVKHKPKATAEQLKTVMDDFAQFLDTCCKAADKDTCFSTEGPNLVTRCKDALA

1 0.070000 Da 0 Trypsin

Carbamidomethyl (C)

138.000000 1 NCBInr gi|26341396

955.528388 0.001090 66 73 0 LCAIPNLR

1298.698174 -0.000099 330 340 0 HPDYSVSLLLR

1438.773061 -0.004924 407 420 0 APQVSTPTLVEAAR

1454.800488 0.001111 329 340 1 RHPDYSVSLLLR

1478.795802 0.007655 390 402 0 LGEYGFQNAILVR

1608.791078 0.008668 316 328 0 DVFLGTFLYEYSR

1661.853909 0.009243 438 451 0 LPCVEDYLSAILNR

1680.838030 0.002138 211 225 0 LSQTFPNADFAEITK

1881.939842 0.010353 477 492 0 RPCFSALTVDETYVPK

1900.903856 0.014848 121 136 0 ENPTTFMGHYLHEVAR

1916.896955 0.013029 121 136 0 ENPTTFMGHYLHEVAR

1980.987743 0.066631 553 570 1 AADKDTCFSTEGPNLVTR

Sequence coverage: 24.1%, MW: 67.0 kDa

**unnamed protein product [Mus musculus] gi|74137565**

MKWVTFLLLLFVSGSAFSRGVFRREAHKSEIAHRYNDLGEQHFKGLVLIAFSQYLQKCSYDEHAKLVQEVTDFAKTCVADESAANCDKSLHTLFGDKLCAIPNLRENYGELADCCTKQEPERNECFLQHKDDNPSLPPFERPEAEAMCTSFKENPTTFMGHYLHEVARRHPYFYAPELLYYAEQYNEILTQCCAEADKESCLTPKLDGVKEKALVSSVRQRMKCSSMQKFGERAFKAWAVARLSQTFPNADFAEITKLATDLTKVNKECCHGDLLECADDRAELAKYMCENQATISSKLQTCCDKPLLKKAHCLSEVEHDTMPADLPAIAADFVEDQEVCKNYAEAKDVFLGTFLYEYSRRHPDYSVSLLLRLAKKYEATLEKCCAEANPPACYGTVLAEFQPLVEEPKNLVKTNCDLYEKLGEYGFQNAILVRYTQKAPQVSTPTLVEAARNLGRVGTKCCTLPEDQRLPCVEDYLSAILNRVCLLHEKTPVSEHVTKCCSGSLVERRPCFSALTVDETYVPKEFKAETFTFHSDICTLPEKEKQIKKQTALAELVKHKHKATAEQLKTVMDDFAQFLDTCCKAADKDTCFSTEGPNLVTRCKDALA

1 0.070000 Da 0 Trypsin

Carbamidomethyl (C)

136.000000 2 NCBInr gi|74137565

955.528388 0.001090 98 105 0 LCAIPNLR

1298.698174 -0.000099 362 372 0 HPDYSVSLLLR

1438.773061 -0.004924 439 452 0 APQVSTPTLVEAAR

1454.800488 0.001111 361 372 1 RHPDYSVSLLLR

1478.795802 0.007655 422 434 0 LGEYGFQNAILVR

1608.791078 0.008668 348 360 0 DVFLGTFLYEYSR

1661.853909 0.009243 470 483 0 LPCVEDYLSAILNR

1680.838030 0.002138 243 257 0 LSQTFPNADFAEITK

1881.939842 0.010353 509 524 0 RPCFSALTVDETYVPK

1900.903856 0.014848 153 168 0 ENPTTFMGHYLHEVAR

1916.896955 0.013029 153 168 0 ENPTTFMGHYLHEVAR

1980.987743 0.066631 585 602 1 AADKDTCFSTEGPNLVTR

Sequence coverage: 22.9%, MW: 70.7 kDa

**unnamed protein product [Mus musculus] gi|26340966**

MKWVTFLLLLFVSGSAFSRGVFRREAHKSEIAHRYNDLGEQHFKGLVLIAFSQYLQKCSYDEHAKLVQEVTDFAKTCVADESAANCDKSLHTLFGDKLCAIPNLRENYGELADCCTKQEPERNECFLQHKDDNPSLPPFERPEAEAMCTSFKENPTTFMGHYLHEVARRHPYFYAPELLYYAEQYNEILTQCCAEADKESCLTPKLDGVKEKALVSSVRQRMKCSSMQKFGERAFKAWAVARLSQTFPNADFAEITKLATDLTKVNKECCHGDLLECADDRAELAKYMCENQATISSKLQTCCDKPLLKKAHCLSEVEHDTMPADLPAIAADFVEDQEVCKNYAEAKDVFLGTFLYEYSRRHPDYSVSLLLRLAKKYEATLEKCCAEANPPACYGTVLAEFQPLVEEPKNLVKTNCDLYEKLGEYGFQNAILVRYTQKAPQVSTPTLVEAARNLGRVGTKCCTLPEDQRLPCVEDYLSAILNRVCLLHEKTPVSEHVTKCCSGSLVERRPCFSALTVDETYVPKEFKAETFTFHSDICTLPEKEKQIKKQTALAELVKHKPKATAEQLKTVMDDFAQFLDTCCKAADKDTCFSTEGPNLVTRCKDTLA

1 0.070000 Da 0 Trypsin

Carbamidomethyl (C)

136.000000 2 NCBInr gi|26340966

955.528388 0.001090 98 105 0 LCAIPNLR

1298.698174 -0.000099 362 372 0 HPDYSVSLLLR

1438.773061 -0.004924 439 452 0 APQVSTPTLVEAAR

1454.800488 0.001111 361 372 1 RHPDYSVSLLLR

1478.795802 0.007655 422 434 0 LGEYGFQNAILVR

1608.791078 0.008668 348 360 0 DVFLGTFLYEYSR

1661.853909 0.009243 470 483 0 LPCVEDYLSAILNR

1680.838030 0.002138 243 257 0 LSQTFPNADFAEITK

1881.939842 0.010353 509 524 0 RPCFSALTVDETYVPK

1900.903856 0.014848 153 168 0 ENPTTFMGHYLHEVAR

1916.896955 0.013029 153 168 0 ENPTTFMGHYLHEVAR

1980.987743 0.066631 585 602 1 AADKDTCFSTEGPNLVTR

Sequence coverage: 22.9%, MW: 70.7 kDa

**alpha-fetoprotein gi|191765**

CAIPNLRENYGELADCCTKQEPERNECFLQHKDDNPSLPPFERPEAEAMCTSFKENPTTFMGHYLHEVARRHPYFYAPELLYYAEQYNEILTQCCAEADKESCLTPKLDGVKEKALVSSVRQRMKCSSMQKFGERAFKAWAVARLSQTFPNADFAEITKLATDLTKVNKECCHGDLLECADDRAELAKYMCENQATISSKLQTCCDKPLLKKAHCLSEVEHDTMPADLPAIAADFVEDQEVCKNYAEAKDVFLGTFLYEYSRRHPDYSVSLLLRLAKKYEATLEKCCAEANPPACYGTVLAEFQPLVEEPKNLVKTNCDLYEKLGEYGFQNAILVRYTQKAPQVSTPTLVEAARNLGRVGTKCCTLPEDQRLPCVEDYLSAILNRVCLLHEKTPVSEHVTKCCSGSLVERRPCFSALT

1 0.070000 Da 0 Trypsin

Carbamidomethyl (C)

96.900000 3 NCBInr gi|191765

1298.698174 -0.000099 264 274 0 HPDYSVSLLLR

1438.773061 -0.004924 341 354 0 APQVSTPTLVEAAR

1454.800488 0.001111 263 274 1 RHPDYSVSLLLR

1478.795802 0.007655 324 336 0 LGEYGFQNAILVR

1608.791078 0.008668 250 262 0 DVFLGTFLYEYSR

1661.853909 0.009243 372 385 0 LPCVEDYLSAILNR

1680.838030 0.002138 145 159 0 LSQTFPNADFAEITK

1900.903856 0.014848 55 70 0 ENPTTFMGHYLHEVAR

1916.896955 0.013029 55 70 0 ENPTTFMGHYLHEVAR

Sequence coverage: 23.2%, MW: 48.8 kDa

0.066631 553 570 1 AADKDTCFSTEGPNLVTR
