# Supplementary material for: Secretogranin II; a Protein Increased in the Myocardium and Circulation in Heart Failure with Cardioprotective Properties
Source: PLoS One. 2012 May 24;7(5):e37401. doi: 10.1371/journal.pone.0037401 (PMC3360055; doi:10.1371/journal.pone.0037401)
Supplement: Supporting Information S2 — Supplemental methods. (DOC) [file pone.0037401.s005.doc]

**Supporting Information S2**

**to**

**Secretogranin II; a protein increased in the myocardium and circulation in heart failure with cardioprotective properties**

**Helge Røsjø MD^1,2^; Mats Stridsberg MD, PhD^3^; Geir Florholmen MSc, PhD^2,4^; Kåre-Olav Stensløkken MSc, PhD^5^; Anett Hellebø Ottesen MSc^1,2,4^**; **Ivar Sjaastad MD, PhD^2,4^; Cathrine Husberg MSc, PhD^2,4^; Mai Britt Dahl MSc^1,2,6^; Erik Øie MD, PhD^2,7^; William E. Louch MSc, PhD^2,4^; Torbjørn Omland MD, PhD, MPH^1,2^; Geir Christensen MD, PhD, MHA^2,4^**

^1^ Division of Medicine, Akershus University Hospital, Lørenskog, Norway

^2^ Center for Heart Failure Research and K.G. Jebsen Cardiac Research Centre, Institute of Clinical Medicine, University of Oslo, Oslo, Norway

^3^ Department of Medical Sciences, Uppsala University, Uppsala, Sweden

^4^ Institute for Experimental Medical Research, Oslo University Hospital, Ullevål, Oslo, Norway

**^5^** Department of Molecular Biosciences, University of Oslo, Oslo, Norway

^6^ Department of Clinical Molecular Biology, Akershus University Hospital, Lørenskog, Norway

^7^ Research Institute for Internal Medicine, Oslo University Hospital, Rikshospitalet, Oslo, Norway

**SUPPLEMENTAL METHODS**

**Mouse model of HF**

Six week old C56BL/6 mice (Taconic, Skensved, Denmark) were used for experiments. Surgical procedures were performed as earlier described with a permanent ligation of the left main coronary artery in the heart failure (HF) group after a left-sided thoracotomy and pericardectomy [1]. Sham-operated animals (sham) were subjected to the same procedure except ligation of the coronary artery. A full echocardiographic examination was performed one week after the primary operation while animals were anesthetized breathing a gas mixture of oxygen and isoflurane supplied via a facemask. Inclusion in the HF group was based on criteria previously validated by our group as sensitive and specific for diagnosing HF: 1) Myocardial infarction >40 % of the circumference of the left ventricle (LV), 2) left atrial diameter >2.0 mm, 3) >35 % increase in lung weight compared to the sham group (e.g. lung weight >0.2 g) [2].

After sacrificing the animals, hearts and the other organs were dissected, prepared, and stored as previously described [1]. In the LV, the thin infarcted region was dissected from the hypertrophic non-infarcted region. To avoid interference of necrotic cardiomyocytes to non-infarcted LV tissue, the transformation zone (border zone) was included in the infarcted region. Tissue for RT-qPCR analysis or immunoblotting were immediately frozen in liquid nitrogen and stored at 70°C until use.

**Quantitative real-time PCR (RT-qPCR)**

RNA was extracted from the non-infarcted region of mice LV tissues using the SV Total RNA Isolation System (Promega Corporation, Madison, WI, USA) as previously described (20-35 mg, 9 HF mice and 8 sham) [1]. From neonatal rat cardiomyocytes, total RNA was isolated with the RNeasy mini kit (Qiagen, Hilden, Germany), RNA concentration and quality was assessed [1], and cDNA produced with the High-Capacity cDNA Archive kit (Applied Biosystems, Foster City, CA, USA) or the iScript cDNA Synthesis Kit (Bio-Rad Laboratories Inc., Hercules, CA, USA). The results were detected on a 7900 HT Real-Time PCR System (Applied Biosystems), and the relative gene expression was determined by the standard curve method [3]. All samples were run in triplicate, RPL4 served as an internal control, and levels are presented as fold change (change from the mean in the sham group) ± SEM.

**Radioimmunoassay on tissue homogenate**

Frozen myocardial tissue samples were homogenized and pre-treated with SDS, and the total protein content measured as previously described [1]. SgII levels were measured by an in-house made region-specific radioimmunoassay (RIA) detecting the secretonurin part of SgII (SgII_154-165_) [4]. No sample had SgII levels below the detection limit (<1 fmol/tube). All samples were measured in duplicate.

**1-D gel electrophoresis and immunoblotting**

We performed 1-D gel electrophoresis and immunoblotting according to standard procedures. Total protein content was measured with the micro BCA protein assay kit (Pierce Biotechnology, Rockford, IL, USA), and the lysates were denaturated for 5 min at 100°C prior to gel loading [1]. We used polyacrylamide gels (10-12 %) and the Precision Plus Protein Dual Color Standard (161-0374) as the molecular marker (Bio-Rad Laboratories Inc.). A Mini Trans-Blot Cell system (Bio-Rad Laboratories Inc.) was used to transfer proteins from gels to Hybond-P PVDF membranes (RPN303F, Amersham Biosciences Europe, Freiburg, Germany). Non-specific binding to the membrane was blocked with 5% dry milk in TBS/1% Tween for 2 h. After an overnight incubation with the primary monoclonal mouse anti-SgII antibody (diluted 1:5000, ab20246, Abcam, Cambridge, UK) at 4°C, the membranes were incubated for 1 h with a secondary goat anti-mouse IgG antibody (1030-50, Southern Biotech, Birmingham, AL, USA). Membranes were washed in between and after all incubation steps with TBS/1% Tween. The primary polyclonal anti-PC1/3 antibody was from Millipore, Billerica, MA, USA (diluted 1:500, AB10553) and the primary polyclonal anti-PC2 antibody was from Santa Cruz Biotechnology, Santa Cruz, CA, USA (diluted 1:100, sc-22891). The secondary antibody for PC1/3 was goat anti-rabbit IgG antibody (4030-05, Southern Biotech) and for PC2 a rabbit anti-goat IgG antibody (6160-05, Southern Biotech). The band at 110 kDa was measured for PC1/3 according to the manufacturer’s specifications [5], while the bands at 75 and 68 kDa were assessed to determine the pro-form and active form of PC2, respectively [6].

We used a similar protocol to assess Stat3, Akt, and Erk1/2 regulation in cardiomyocytes after secretoneurin stimulation. Membranes were first incubated with phosphospesific antibodies against Stat3 (Tyr_705_ or Ser_724_), Akt (Ser_473_), and Erk1/2 (_Thr202/Tyr204_), then stripped, before being reprobed with antibodies against total Stat3, Akt and Erk1/2 for equal protein loading (all antibodies from Cell Signaling Technologies, Beverly, MA, USA). Levels of phosphorylated protein forms were normalized to the corresponding total level of Stat3, Akt or Erk1/2. Equal protein loading on gels was controlled by using anti-glyceraldehyde-3-dehydrogenase (GAPDH) as an internal control (2118, Cell Signaling Technology). We used a secondary anti-goat antibody for GADPH analysis (4030-05, Southern Biotech).

**Mass spectrometry peptide mass fingerprinting**

Coomassie stained protein bands were cut out from SDS-polyacrylamide gels and the proteins were in-gel reduced by dithiothreitol (DTT), alkylated by iodoacetamide (IAA), and digested by trypsin. Obtained peptides were purified on C18-stop and go extraction tips (C18 Empore Extraction Disks, Varian, St. Paul, MN, USA) [7], placed in GELoader tips (Eppendorf, Hamburg, Germany) and eluted directly onto a stainless-steel target plate by acetonitrile (HPLC grade S, Rathburn Chemicals, Walkerburn, Scotland)/H_2_O (70:30, v/v) containing 10 mg/mL α-cyano-4-hydroxycinnamic acid (Sigma-Aldrich) and 0.1% trifluoroacetic acid (Fluka, Buchs, Switzerland). After crystallization of the eluted samples, the peptides were analyzed on an Ultraflex II matrix-assisted laser desorption/ionization time of flight (MALDI-TOF/TOF) mass spectrometer (Bruker Daltonics, Bremen, Germany) operated in the positive reflector mode. Mass lists obtained from the acquired mass-spectra were further submitted for database searching (NCBI) by the Mascot search engine (Matrix Science Ltd, London, UK) to identify the proteins.

**Immunohistochemistry**

The distribution of SgII in the myocardium was examined in 3 mice with HF. Hearts were removed, fixed overnight in 4% formalin, washed in 30% ethanol and stored in 70% ethanol at 4°C before use. We used a SgII antibody binding to the C-terminal region of secretoneurin (SgII_172-186_) [8] that were followed by anti-goat IgG (Vector Laboratories, Burlingame, CA, USA). The avidin-biotin-peroxidase system (Vectastain Elite kit, Vector Laboratories) was used to further amplify immunoreactivity, before sections were counter-stained with hematoxylin [1]. To investigate unspecific staining, we used non-immune rabbit serum or omitted the primary antibody.

**Langendorff perfusion**

Male Wistar rats (250-350 g, Scanbur AS, Nittedal, Norway) were used for these experiments. Animals were acclimatized for at least four days before any experiments were conducted.

The heart temperature was kept constant during the experiment by the surrounding glass tube (inner diameter 40 mm, height 80 mm) perfused with water from the heating chamber. A fluid-filled latex balloon was inserted into the LV to measure ventricular pressures by a Powerlab system (AD Instruments Pty Ltd, Castle Hill, NSW 2154, Australia). LV end-diastolic pressure (LVEDP) was set to 5-10 mmHg and changes in LVEDP were measured. Myocardial temperature was controlled by inserting a temperature probe in the right ventricle. The hearts with LV systolic pressure ≤ 100 mmHg, coronary flow ≤ 8 or ≥ 20 mL/min, heart rate ≤ 220 beats per minute before ischemia, or irreversible arrhythmias for more than 30 min during reperfusion were excluded from the study. After 120 min of reperfusion, the hearts were cut in four slices of 1 mm and three slices of 2 mm (hearts fixed in acrylic rat brain matrix by AgnThor´s AB, Lidingö, Sweden). The 2 mm slices were freeze clamped in liquid nitrogen and stored for later analyses, while the other slices collected 5-8 mm from apex were incubated in 1% triphenyltetrazoliumchloride for 15 min at 37ºC. After incubation the slices were gently pressed between two glass plates and photographed (Nikon, Colorfix5400, Tokyo, Japan).

**Cell culture experiments**

To explore endocrine and paracrine factors associated with SgII production, neonatal (1-3 days) Wistar rats (Taconic) were used for extraction of cardiomyocytes as previously reported [9]. Total RNA was isolated, quality assessed, and mRNA levels determined as described above. To assess the short-term effect of secretoneurin stimulation on phospho proteins, the cardiomyocytes were stimulated for 10 or 30 min with 10 µg/mL secretoneurin (NeoMPS, Strasbourg, France) or vehicle. Cells were harvested in lysis buffer (Tris pH 7.6, 5M NaCl, 0.5M EDTA, 0.1M EGTA, 1M B-gly [Sigma-Aldrich St. Louis, MO, USA] and NP-40), and the protein levels measured by Western blotting as described above.

We isolated cardiomyocytes from the LV by retrograd perfusion *ex vivo* with collagenase (Collagenase type II, Worthington Biochem. Corp., Lakewood, NJ, USA) in sham rats [10]. Cardiomyocytes were separeted from non-cardiomyocytes by differential centrifugation [11]. Immunocytochemical analysis confirmed that >95% of the cells in the cardiomycoyte fraction were sarcomeric actin-positive cardiomyocytes [10].

**Patients with HF and healthy control subjects**

Inclusion of HF patients and healthy control subjects were performed as previously described [1]. For this study we only included stable HF patients, e.g. patients not hospitalized for worsening of HF during the last three months prior to study commencement. A transthoracic echocardiogram had been obtained within the last 18 months in 55 of the 58 patients (95%). The last 3 patients had been clinically stable during this period as evaluated by no change in severity of HF symptoms or New York Heart Association (NYHA) functional class in the last 12 months, and no hospitalization for worsening HF in the last 24 months. One of these patients had a LV ejection fraction of 20% and no evidence of reversible myocardial ischemia when evaluated by myocardial single photon emission computed tomography (SPECT) during the last year prior to study inclusion. Fifty-four patients (93%) had undergone coronary angiography for diagnostic purposes. New York Heart Association (NYHA) functional class were determined by one investigator (HR). All patients were included from Akershus University Hospital's HF outpatient clinic.

Blood samples were processed as previously described [1], and plasma B-type natriuretic peptide (BNP) levels measured by a two-step sandwich immunoassay (Architect® BNP assay, Abbott Diagnostics, Abbott Park, IL, USA). Plasma CgA levels were measured by a commercial RIA identifying CgA_116-439_ (Euro-Diagnostica AB, Malmö, Sweden) and CgB levels assessed by a region-specific RIA detecting CgB_439-451_ [12].

**SUPPLEMENTAL REFERENCE**S

(1) Røsjø H, Husberg C, Dahl MB, Stridsberg M, Sjaastad I, et al (2010) Chromogranin B in heart failure: a putative cardiac biomarker expressed in the failing myocardium. Circ Heart Fail 3:503-511.

(2) Finsen AV, Christensen G, Sjaastad I (2005) Echocardiographic parameters discriminating myocardial infarction with pulmonary congestion from myocardial infarction without congestion in the mouse. J Appl Physiol 98:680-689.

(3) Yuan JS, Reed A, Chen F, Stewart CN, Jr (2006) Statistical analysis of real-time PCR data. BMC Bioinformatics 7:85.

(4) Stridsberg M, Eriksson B, Janson ET (2008) Measurements of secretogranins II, III, V and proconvertases 1/3 and 2 in plasma from patients with neuroendocrine tumours. Regul Pept 148:95-98.

(5) http://www.millipore.com/coa.nsf/a73664f9f981af8c852569b9005b4eee/7eab5eae78c

15145882578960054885a/$FILE/AB10553_NG1865059.pdf (14 Oct 2011)

(6) Berman Y, Mzhavia N, Polonskaia A, Furuta M, Steiner DF, et al (2000) Defective prodynorphin processing in mice lacking prohormone convertase PC2. J Neurochem 75:1763-1770.

(7) Rappsilber J, Ishihama Y, Mann M (2003) Stop and go extraction tips for matrix-assisted laser desorption/ionization, nanoelectrospray, and LC/MS sample pretreatment in proteomics. Anal Chem 75:663-70.

(8) Stridsberg M, Grimelius L, Portela-Gomes GM (2008) Immunohistochemical staining of human islet cells with region-specific antibodies against secretogranins II and III. J Anat 212:229-234.

(9) Florholmen G, Andersson KB, Yndestad A, Austbø B, Henriksen UL, et al (2004) Leukaemia inhibitory factor alters expression of genes involved in rat cardiomyocyte energy metabolism. Acta Physiol Scand 180:133-142.

(10) Øie E, Sandberg WJ, Ahmed MS, Yndestad A, Lærum OD, Attramadal H, et al (2010) Activation of Notch signaling in cardiomyocytes during post-infarction remodeling. Scand Cardiovasc J 44:359-66.

(11) De Young MB, Giannattasio B, Scarpa A (1989) Isolation of calcium-tolerant atrial and ventricular myocytes from rat adult heart. Methods Enzymol. 173:662-76.

(12) Stridsberg M, Eriksson B, Öberg K, Janson ET (2005) A panel of 13 region-specific radioimmunoassays for measurements of human chromogranin B. Regul Pept 125:193-199.
